# Polystyrene microplastics are internalized by human gingival fibroblasts, enhance cell motility and induce molecular changes revealed through proteomic analysis

**DOI:** 10.1038/s41598-025-19064-w

**Published:** 2025-10-08

**Authors:** Federica Di Cintio, Anna Giulia Ruggieri, Chiara De Simone, Piero Di Carlo, Maurizio Ronci, Vittoria Perrotti, Michele Sallese

**Affiliations:** 1 Department of Oral, Medical and Biotechnological Sciences, University “G. d’Annunzio” of Chieti-Pescara, Chieti, Italy; 2 Center for Advanced Studies and Technology (CAST), University “G. d’Annunzio” of Chieti-Pescara, Chieti, Italy; 3 Department of Innovative Technologies in Medicine and Dentistry, University “G. d’Annunzio” of Chieti-Pescara, Chieti, Italy; 4 UdA-TechLab, Research Center, “G. d’Annunzio” University of Chieti-Pescara, Via dei vestini, 31-66100 Chieti, Italy

**Keywords:** Human gingival fibroblast, Microplastics, Polystyrene, EMT, Proteomics, Cell motility, Biochemistry, Cell biology, Proteomic analysis

## Abstract

Plastics pose a significant global threat to the ecosystems due to their accumulation and impact on human health. The degradation of plastics results in the formation of microplastics (MPs), small particles less than 5 mm in size, which are released into the environment. Polystyrene (PS) is a major chemical component of plastics, and exposure to PS-based microplastics (PS-MPs) has been linked to cellular damage. This study aimed to explore the effects of 1 μm PS-MPs on human gingival fibroblast cells (hGF). PS-MPs induced a limited cytotoxicity at the tested concentrations and time points. Confocal microscopy, TEM and flow cytometry revealed the uptake of PS-MPs in about 10% of hGF cells. Proteomics identified a profoundly altered proteostasis in PS-MPs treated cells. Enrichment analysis of differentially expressed proteins uncovered disruptions in cellular pathways, including metabolisms (glycolysis and adipogenesis), endocrine functions (androgen and oestrogen responses), inflammatory responses (interferons α and γ) and cancer progression (epithelial-mesenchymal transition). Consistent with the enrichment analysis, treated cells exhibited a significant increase in motility. This study demonstrated that PS-MPs are internalized by cells and influence key cellular pathways related to inflammation, motility, and survival, reinforcing the notion that PS-MP exposure may pose risks to human health.

**Supplementary Information:**

The online version contains supplementary material available at 10.1038/s41598-025-19064-w.

## Introduction

Plastics have been recognized by the international scientific community as an emerging global threat to the environment and human health due to their widespread accumulation into air, water, and soil. Their versatility and chemical stability make them extensively used as packaging materials, disposable items, foodservice products, construction materials, and consumer goods^1,2^. However, inadequate recycling policies have contributed to significant environmental pollution, raising global concerns about their impact on human health^3^. Plastics are highly persistent into the environment^4,5^ due to their inert nature, making them difficult to decompose. Degradation occurs via chemical weathering, photo-oxidation, and environmental fragmentation, ultimately generating microplastics (MPs) (< 5 mm in diameter)^6,7^ and further breaking down into nanoplastics (NPs < 1000 nm)^6,8–12^. Furthermore, MPs are also intentionally manufactured as microbeads for use in cosmetics, cleaning products, fertilizers, and as raw materials in plastic production. Indeed, the term “microplastic” encompasses a wide range of polymeric materials of varying sizes, shapes (e.g. spheres, fibres, fragments), and chemical formulations^13^.

Overall the ubiquitous diffusion of MPs have dramatically increased the risk of ecosystem contamination, making their potential effects on human health a growing area of interest^5^. Among the most prevalent polymeric components of MPs is polystyrene (PS), a non-biodegradable synthetic aromatic hydrocarbon polymer derived from styrene monomers^2^. PS-MPs can interact with various organic and inorganic environmental pollutants, including heavy metals, organic matter, or microorganisms, further exacerbating their toxicological potential^14^.

Humans are exposed to MPs and NPs through multiple routes of entry^8,15^including ingestion, inhalation, skin contact, and oral exposure. Among these, the upper respiratory tract, especially the oral mucosa, serves as the first biological barrier against airborne pollutants potentially leading to systemic damage in various organs^16–20^. The gingival area, in particular, can retain food particles—and potentially MPs—due to anatomical features like the gingival sulcus and periodontal pockets, increasing the chance of local cellular interaction^21,22^. Additionally, dental devices such as clear aligners and occlusal splints, made of plastic and worn for extended periods, may release micro- and nanoplastics over time^23^. These factors make gingival cells a relevant model for studying MP exposure.

PS-MPs can penetrate biological membranes, accumulate in tissues and organs, and induce oxidative stress through the production of reactive oxygen species (ROS)^24^. Caputi et al., demonstrated that the exposure to MPs in human gingival fibroblast (hGF) activates the NFkB/MyD88/NLRP3 inflammatory pathway, leading to increased expression of inflammatory markers both at gene and protein levels (NFkB, MyD88, and NLRP3)^25^. Other researchers also investigated the toxic effects of PS-MPs in mice intestines, revealing that exposure to 50 nm and 500 nm PS-MPs induced toxicity by ROS-mediated apoptosis in epithelial cells^26^. Moreover, Lu et al., demonstrated 100 nm and 500 nm PS-NPs exhibit low acute toxicity in human umbilical vein endothelial cells (HUVEC)^27^. Xiao et al. used a kidney-testicle microfluidic platform (KTP) to reveal that PS-NPs enter the kidneys and testes through endocytosis, dysregulating key cancer-related pathways, such as MAPK and PI3K-AKT signaling^24^. Finally, Fan et al., investigated the effects of 100 nm and 1 μm PS-MPs in rat lungs, revealing alveolar and bronchial epithelial damage and increased expression of pro-inflammatory cytokines IL-6, TNF-α, and IL-1β. Moreover, novel circular RNAs and non-coding RNAs were identified as potential mediators of PS-MPs-induced lung inflammation^28^.

While the impact of MPs has been extensively investigated in the lower respiratory and gastrointestinal tracts, their effects in the upper respiratory and digestive tracts remain largely unexplored, despite direct exposure to inhaled MPs.

In the present study, we investigated the effects of PS-MPs on hGF by analysing PS-MP cytotoxicity, internalization, differential protein expression, and cell migration. We found that PS-MPs are not cytotoxic at concentrations ranging from 0.25 µg/mL to 25 µg/mL, while a slight cytotoxicity was observed when cells were treated with 50 µg/mL of PS-MPs. The PS-MPs were endocytosed efficiently by a small percentage of the cells and mainly accumulated into endosomes. Proteomic analysis identified 389 differentially expressed proteins involved in key pathways that impact metabolism, inflammation, endocrine functions and cell migration. Finally, functional studies demonstrated an increase in cell motility following treatment with PS-MPs.

## Materials and methods

### Cell culture conditions

The hGF cell line (Cell Lines Service GmbH, Eppelheim, Germany) was cultured following the supplier in Dulbecco’s Modified Eagle’s Medium (DMEM)/Nutrient Mixture F-12 Ham (Corning, NY, USA). The culture medium was supplemented with 5% heat-inactivated Fetal Bovine Serum (FBS) and 1% Penicillin-Streptomycin (Thermo Fisher Scientific, MA, USA).

### Microplastics

Commercial 1 μm PS-MPs (#S-89904, Sigma-Aldrich, MO, USA) and amine-modified fluorescent PS-MPs 1 μm beads (#L1030, Sigma-Aldrich, MO, USA) were purchased and used as supplied from the vendor.

### Cell metabolic activity

hGFs were seeded at a density of 2 × 10^3^ cells/well into a 96-well tissue culture plate and incubated for 24 h to allow cells attachment. hGFs viability was assessed using MTT assay (3-(4,5-dimethyl-2-thiazolyl)-2,5-diphenyl-2 H-tetrazolium bromide) (#ab146345, Abcam, Cambridge, UK) after 24, 48, and 72 h of treatment with 50, 25, 5, 2.5, and 0.25 µg/mL of PS-MPs, and using vehicle treated cells as control. A volume of 10 µL/well of MTT dye solution was added to the culture medium, and cells were incubated for 3 h at 37 °C. To allow formazan formation, after 3 hours (h) of incubation at 37 °C with the MTT solution on the cells, we added 100 µL/well of dimethyl sulfoxide (DMSO, Sigma-Aldrich, MO, USA) to dissolve the formazan crystals, which produced a purple color. Formazan formation, which is directly proportional to viable cell numbers, was quantified by measuring absorbance at 570 nm using a Synergy™ HT Multi-detection microplate reader (Biotech, Winooski, VT, USA). The MTT assay was performed in three independent experiments. The absorbance reading from the cell-only condition was subtracted from the DMSO-only reading and used as the blank. The untreated condition was used to normalize the readings.

### Cell cycle evaluation

Cell cycle phases were analysed using flow cytometric with propidium iodide (PI) DNA staining (#P1304MP, Invitrogen, MA, USA). hGFs were seeded at a density of 2.5 × 10^5^ cells/well into a 6-well culture plate and incubated for 24 h to allow attachment. Cells were then treated with 50 µg/mL of PS-MPs for 24, 48, and 72 h. After treatment, cells were harvested, washed with phosphate buffered saline (PBS), fixed in 70%ethanol, and incubated over-night at + 4 °C with PI solution (PBS, PI, RNase A #A797C Promega, Madison, WI, USA). A total of 10.000 events/sec were acquired using a BD FACS Canto II flow cytometry (BD Bioscience, Franklin Lakes, NJ), and data were analyzed with FLOWJO software v10.10 (BD Bioscience, Franklin Lakes, NJ).

### Confocal fluorescent microscopy analysis

Immunofluorescence (IF) microscopy was used to evaluate the localization of fluorescent PS-MPs in hGF cells. Cells were seeded on coverslips at a density of 2 × 10^4^ and incubated for 24 h to enable cells adhesion. After exposure to 50 µg/mL fluorescent PS-MPs for 24, 48, and 72 h, cells were fixed with 4% paraformaldehyde (PFA) in PBS (Electron Microscopy Sciences, PA, USA) for 10 min. To block non-specific binding, cells were incubated for 1 h with a homemade blocking buffer (PBS with 1% bovine serum albumin (BSA) and 0.01% saponin) (Sigma-Aldrich, MO, USA). Samples were then incubated overnight at 4° C with a primary antibody against early endosomes (EEA1 #610457, BD, Franklin Lakes, NJ, USA), washed three times with PBS, and further incubated with a secondary fluorescent antibody (α-mouse Alexa Fluor 546 #A1135 Invitrogen, MA, USA) for 1 h at room temperature (RT). Finally, coverslips were mounted using Mowiol 4–88 reagent (Merck Millipore, MA, USA), and images were acquired using a ZEISS LSM 800 confocal microscope (ZEISS, Jena, Germany) at 63× magnification. The image analysis was performed with Fiji ImageJ software (National Institutes of Health, MD, USA).

### PS-MPs internalization by flow cytometry

hGFs were seeded at a density of 2.5 × 10^5^ cells/well cell per well in a 6-well culture plate and incubated for 24 h to allow attachment. After treatment with 50 µg/mL fluorescent PS-MPs, the cells were washed twice with PBS and then resuspended in 300 µL of PBS. Microplastic internalization was quantified at 24, 48, and 72 h using flow cytometry, by measuring green fluorescent signals. A total of 10.000 events/sec were acquired using BD FACS Canto II flow cytometer (Beckton Dickinson), and data were analysed by FLOWJO software v10.10 (BD Bioscience, Franklin Lakes, NJ).

### Transmission electron microscopy (TEM) analysis and imaging

For TEM analysis, hGFs were seeded at a density of 10 × 10^4^ cells/well in a 35 mm uncoated dish (#P35G-1.5-14-C, MatTek, Bratislava II Slovak Republic). Cells were treated with 50 µg/mL PS-MPs for 48, and 72 h, and vehicle-treated as control. After treatment, cells were fixed in modified Karnosky’s fixative (4%PFA, 1% glutaraldehyde in 0.1 M cacodylate buffer) for 30 min at RT, followed by a 48 h fixation at 4°C in diluted fixative (1:10 in 0.1 M cacodylate buffer). Samples were then post-fixed with 1% osmium tetra-oxide (OsO_4_) (EMS, Hatfield, PA, USA) and 1% potassium ferrocyanide for 1 h at 37 °C, followed by 1% uranyl acetate staining for 30 min at RT. Following sequential dehydration in a graded series of ethanol solutions, samples were embedded in Hard-Plus epoxy resin (EMS, Hatfield, PA, USA) and polymerized at 60 °C for 16 h.

Thin sections (~ 70 nm) were obtained using a UC6 ultramicrotome (Leica, Milan, Italy) and visualized using a Talos L120C transmission electron microscope (Thermo Fisher Scientific, MA, USA) at 120 kV, with magnifications ranging from 2,000× to 53,000×. Images were captured using a 4 K × 4 K Gatan CCD camera (Gatan Inc., Pleasanton, CA, USA).

### Shotgun proteomics

hGFs were seeded at a density of 2 × 10^5^ cells/well into a 6-well tissue culture plate and incubated for 24 h to allow cell attachment. Cells were treated with 50 µg/mL of PS-MPs or vehicle for 48 and 72 h, after which they were collected and lysed in RIPA buffer (50 Tris-HCl (pH 7.6), 0.1% sodium deoxycholate, 0.1% SDS, 1% Triton-X-100,140 mM NaCl), supplemented with protease and phosphatase inhibitors. The Pierce™ BCA Protein Assay Kit (Thermo Fisher Scientific, MA, USA) was used to determine total protein concentration. In these experiments, the untreated control was sampled at 48 h. However, we also analyzed proteome changes between untreated cells at 48 and 72 h to assess temporal variability. These controls were highly similar, with only a few proteins differentially expressed between the two time points—an outcome that falls within the expected variability of the method (data not shown). A total of 25 µg of proteins was loaded into a Nanosep 10-kDa-cutoff filter (Pall Corporation, Michigan, USA) and digested following previously reported protocols^29,30^. Briefly, samples were washed twice with 200 µL urea buffer (8 M urea, 100 mM Tris pH 8.5 in milliQ water) to remove salts and detergents. Proteins were reduced by adding 100 µL of DTT solution (8 mM DTT in urea buffer) and alkylated with 100 µL of Iodoacetamide (IAA) solution (50 mM IAA in urea buffer). The urea buffer was then exchanged with 50 mM ammonium bicarbonate, followed by trypsin digestion at a 1:50 (enzyme: proteins ratio, incubated overnight at 37 °C. The peptide mixture was collected by centrifugation, acidified with 10% trifluoroacetic acid, and stored at -20 °C until analysis. Each digested protein sample was analysed in technical triplicate by LC-MS/MS on a UltiMate3000 RSLCnano (Thermo Fisher Scientific, Waltham, MA, USA) chromatography system coupled to an Orbitrap Fusion Tribrid mass spectrometer, operating in positive ionization mode, equipped with a nanoESI source (EASY-Spray NG) (Thermo Fisher Scientific, Waltham, MA, USA). In detail, peptides were loaded on a PepMap100 C_18_ pre-column cartridge (5 μm particle size, 100 Å pore size, 300 μm i.d. × 5 mm length, Thermo Fisher Scientific, Waltham, MA, USA), and separated on an EASY-Spray PepMap RSLC C_18_ column (2 μm particle size, 100 Å pore size, 75 μm i.d. × 15 cm length, Thermo Fisher Scientific, Waltham, MA, USA) at a 300 nL/min flow rate and a 35 °C temperature. The chromatographic gradient was set as follows: from 95% eluent A (0.1% Formic Acid in water) to 35% eluent B (99.9% Acetonitrile, 0.1% Formic Acid) in 45 min and a total LC run of 65 min. Precursor (MS1) survey scans were recorded in the Orbitrap, at resolving powers of 120 K (at m/z 200). Data-dependent MS/MS (MS2) analysis was performed in top speed mode with a 3 s cycle time, during which the most abundant multiple-charged (2+–7+) precursor ions detected within the range of 375–1500 m/z were selected for activation in order of abundance and detected in ion trap at rapid scan rate. Quadrupole isolation with a 1.6 m/z isolation window was used, and dynamic exclusion was enabled for 60 s after a single scan. Automatic gain control targets were 4.0 × 10^5^ for MS1 and 2.0 × 10^3^ for MS2, with 50 and 300 ms maximum injection times, respectively. For MS2, the signal intensity threshold was 5.0 × 10^3^, and the option “Injection Ions for All Available Parallelizable Time” was set. High-energy collisional dissociation (HCD) was performed using 30% normalized collision energy.

### Bioinformatics

Raw data were processed using PEAKS studio Xpro (Bioinformatics Solutions Inc., Waterloo, Ontario, Canada) using the ‘correct precursor only’ option enabled. Spectra were matched against the UniProt SwissProt database - restricted to Homo sapiens taxonomy – supplemented with a list of common contaminants (20,619 entries). The false discovery rate (FDR) was set to 0.5% at the peptide level. The post-translational modification (PTM) profile was set as follows: fixed cysteine carbamidomethylation (ΔMass: 57.02), variable methionine oxidation (ΔMass: 15.99). Non-specific cleavage was allowed to one end of the peptides, with a maximum of 2 missed cleavages and trypsin enzyme specificity. The highest error mass tolerances for precursors and fragments were set at 10 ppm and 0.5 Da, respectively.

After processing every single raw data, the label free quantification (LFQ) tool of PEAKS Studio was used to detect differentially expressed proteins. Parameters for LFQ were set as follows. Quantification type: Label free quantification; Mass Error Tolerance: 10.0 ppm; Retention Time Shift Tolerance: 0.5 min; FDR Threshold: 0.5%. Statistical significance of differentially expressed proteins was assessed by ANOVA using a threshold at the protein level ≥ 20 − 10 lgP, a fold change ≥ 1.3 and at least 1 peptide for quantification.

Bioinformatic analysis was performed using the BigOmics Playground platform (https://bigomics.ch/) version v3.3.4.9001-master240426. Proteomic datasets from PEAKS Studio LFQ were uploaded into the platform, selecting “homo sapiens” taxonomy and selecting t-test and trend.limma for Gene testing and gsva and fisher for Geneset testing.

### Western blot analysis

Total protein extracts were obtained by lysing the cells with RIPA buffer (50 Tris-HCl (pH 7.6), 0.1% sodium deoxycholate, 0.1% SDS, 1% Triton-X-100, 140 mM NaCl) supplemented with protease and phosphatase inhibitors (Merk, Darmstadt, Germany). After centrifugation at 12,000 rpm for 20 min at 4 °C, an equal amount of protein (25 µg) was separated using Precast 8–16% Gel (GenScript, Piscataway, NJ, USA). Proteins were transferred onto nitrocellulose membrane, blocked for 1 h with 5% BSA and 1% non-fat dried milk in Tris-Buffered Saline (TBS), and incubated overnight at 4 °C with primary antibodies: α-fibronectin (#F3648, Sigma-Aldrich, MO, USA), α-STAT1 (#9172, Cell Signaling, MA, U.S.A), α-Calreticulin (#SPA-601, Enzo Life Sciences, Inc., NY, U.S.A), α-VDAC1 (#PA1954A, Invitrogen, MA, USA), and α-β-actin (#sc-69879, Santa Cruz, CA, USA). After three washes with TBS-T (0.1% TWEEN-20 in TBS), membranes were incubated for 1 h at RT with HRP-conjugated secondary antibodies (α-rabbit and α-mouse HRP IgG H&L Calbiochem, Merck Millipore, Burlington, MA, USA). Chemiluminescent detection was performed using ECL Star Enhanced Chemiluminescent Substrate (Euroclone, Milan, Italy) and bands were visualized using the UVITEC imaging system and software (UVITEC, Cambridge, UK). Densitometric quantification was performed using Fiji ImageJ software (National Institutes of Health, MD, USA).

### Migration assay

Cell motility was evaluated using the Sartorius Incucyte S3 live cell analysis system. hGF cells (7 × 10^3^ cells/well) were seeded into 96-well plates and incubated for 24 h to allow cell attachment. A confluent cell monolayer was scratched using a WoundMaker™ (Essen Bioscience, Ann Arbor, MI, USA), washed with PBS, and incubated with a standard growth medium containing 50 µg/mL PS-MPs or vehicle. Phase-contrast images were captured immediately after scratching using a 10x objective, and subsequently every 3 h up to 72 h. The migration rate was determined by measuring the percentage of wound closure over time using IncuCyte ZOOM^®^ Software.

### Statistical analysis

All statistical analyses were performed using GraphPad Prism v9.01 (GraphPad Software, San Diego, CA, USA). Data are expressed as mean ± standard deviation (SD). Each experiment was repeated at least three times. Shapiro-Wilk test was used to assess the Normality of the distributions. After confirming the normal distributions, MTT assay data were analysed using one-way ANOVA with Sidak’s multiple comparisons. Western blot data were analysed by one-way ANOVA. The wound healing assay data were analysed by two-way ANOVA with Sidak’s multiple comparisons. Statistical significance was set at *p* < 0.05, and error bars represent the standard deviation (SD).

## Results

### PS-MPs exhibit cytotoxic effects on hGF cells

**Fig. 1:**
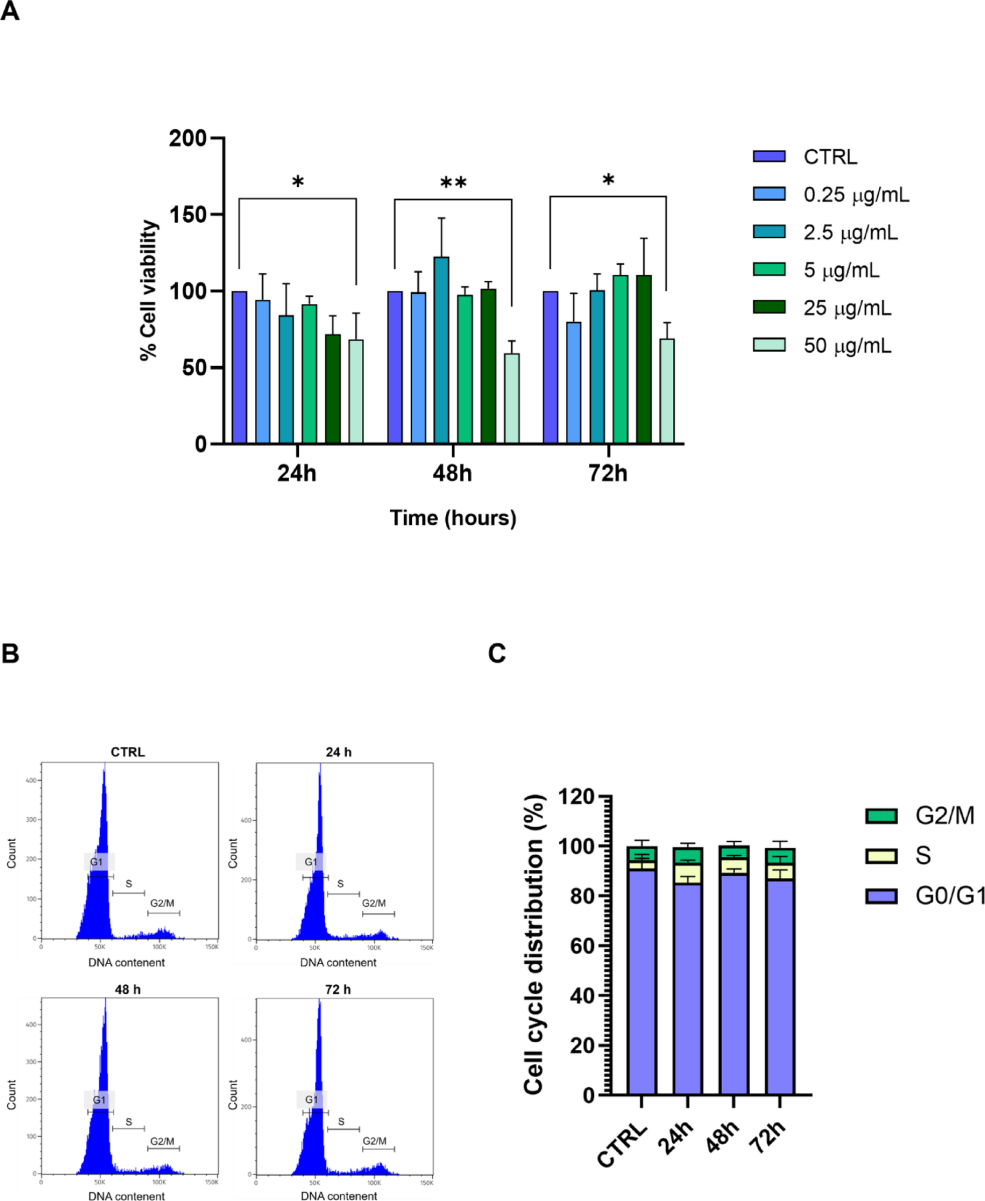
Cytotoxic effect in hGF cells following PS-MPs treatment. (**A**) hGF cells were treated with increasing concentrations of PS-MPs (0.25, 2.5, 5, 25, 50 µg/mL) or left untreated (CTRL). Cell viability is expressed as % of viable cells relative to the control (* *p*-value < 0.05). (**B**) Flow cytometry analysis of the cell cycle in hGF cells treated with 50 µg/mL PS-MPs and untreated (CTRL) at 24, 48, and 72 h. (**C**) Quantitative analysis of the proportion of hGF cells across different cell cycle phases and treatments.

To evaluate the cytotoxic effect of 1 μm-sized PS-MPs on cell viability, hGF cells were incubated with different concentrations of PS-MPs (0.25, 2.5, 5, 25, and 50 µg/mL) for 24, 48, and 72 h. Untreated cells were used as controls. As shown in Fig. 1A, treatment with PS-MPs did not impact cell vitality at any tested concentration after 24 h. In contrast, cell vitality was significantly reduced after 48 and 72 h of treatment at the concentration of 50 µg/mL, therefore this concentration was selected for further experiments. The effect of PS-MP treatment on the cell cycle, assessed via flow cytometry, showed no significant changes in the percentage of hGF cells in specific cell cycle phases compared to the controls at any of the analysed time points (Fig. 1B, C).

### PS-MPs are internalized by hGF cells

**Fig. 2:**
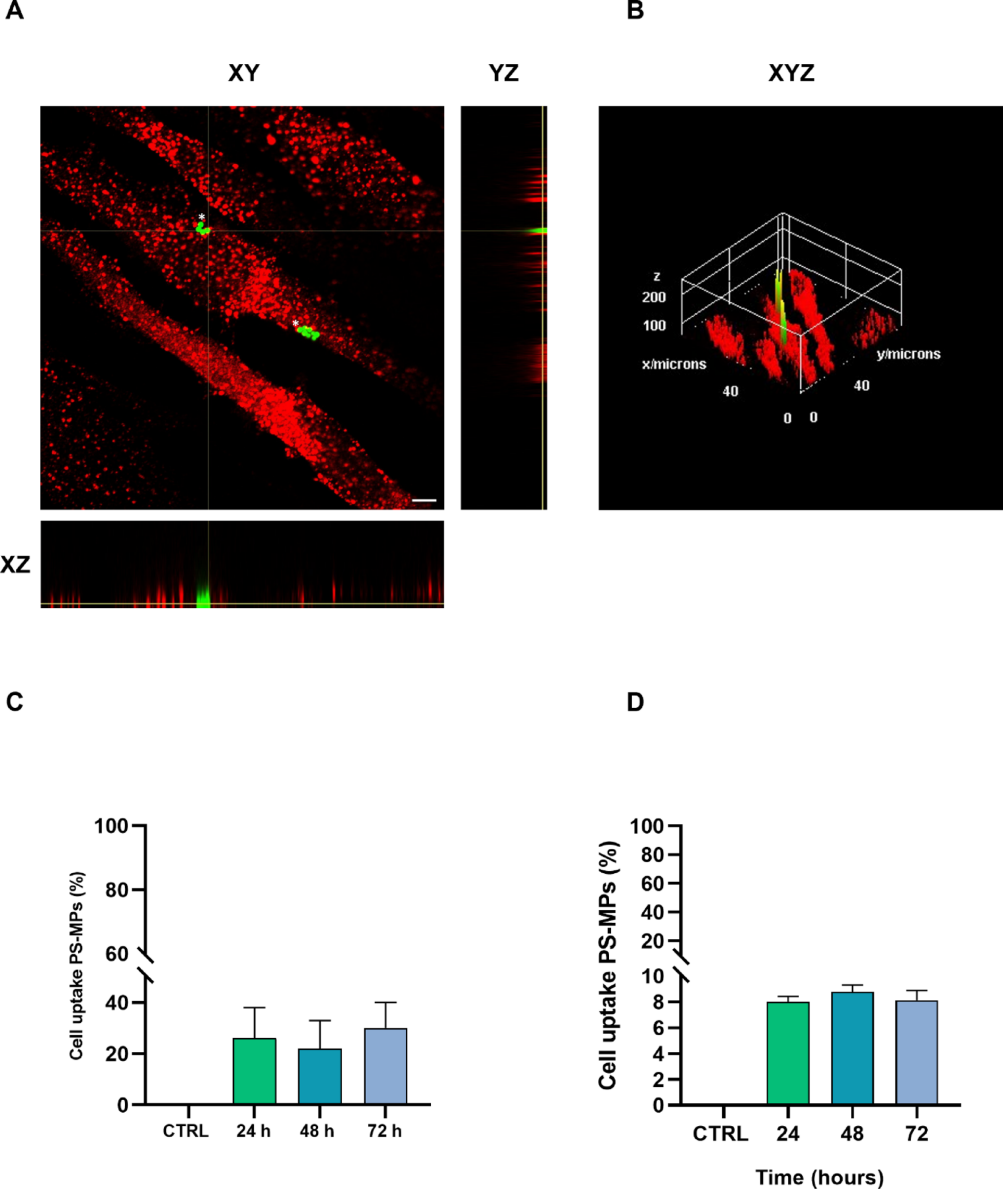
Representative images of PS-MPs uptake by hGF cells. (**A**) CLSM image of hGF cells exposed to 50 µg/mL of 1 μm green-fluorescent PS-MPs for 48 h. Similar images were obtained when cells were treated for either 24–72 h. hGF cells were fixed and stained for the early endosomal marker EEA1 (red). The images are composites from a Z-stack, with sections in the x–y plane (upper left), x–z plane (lower), and y–z plane (upper right). The internalization of PS-MPs is evident in both the ortho YZ plane and XZ plane. Images are representative of three fields per condition (*n* = 3). Scale bars represent 10 μm. (**B**) 3D reconstruction of CLSM images of hGF cells exposed to 50 µg/mL of PS-MPs for 48 h. (**C**) The graph shows the percentage of cells internalizing MPs, based on confocal analysis presented in panels A and B. (**D**) Flow cytometry analysis of PS-MPs uptake in hGF cells. The histogram represents the mean fluorescence intensity of 50 µg/mL of 1 μm green-fluorescent PS-MPs internalized in hGF cells after 24, 48, and 72 h.

To investigate the potential uptake of PS-MPs by hGF cells, confocal laser scanning microscopy (CLSM) experiments were conducted on cells treated with 50 µg/mL of 1 μm green-fluorescent PS-MPs for 24, 48, and 72 h. As shown in Fig. 2, these experiments revealed partial colocalization of fluorescent PS-MPs with the endocytic marker EEA1. Measurements in the x–y plane, conducted at approximately half the cell’s height along the z-axis, confirmed the intracellular presence of PS-MPs at all tested treatment time points (Fig. 2A, B). Figure 2B shows a 3D reconstruction of the cell, further supporting the uptake of PS-MPs. Specifically, manual counting showed that approximately 20% of the cells internalised MPs at each time point (Fig. 2C). Flow cytometry analysis carried out with green-fluorescent PS-MPs confirmed their internalization in hGF cells (Fig. 2C) and demonstrated comparable uptake levels across all tested time points (24 h = 8.00% ± 0.436; 48 h = 8.76% ± 0.551; 72 h = 8.13% ± 0.757).

**Fig. 3:**
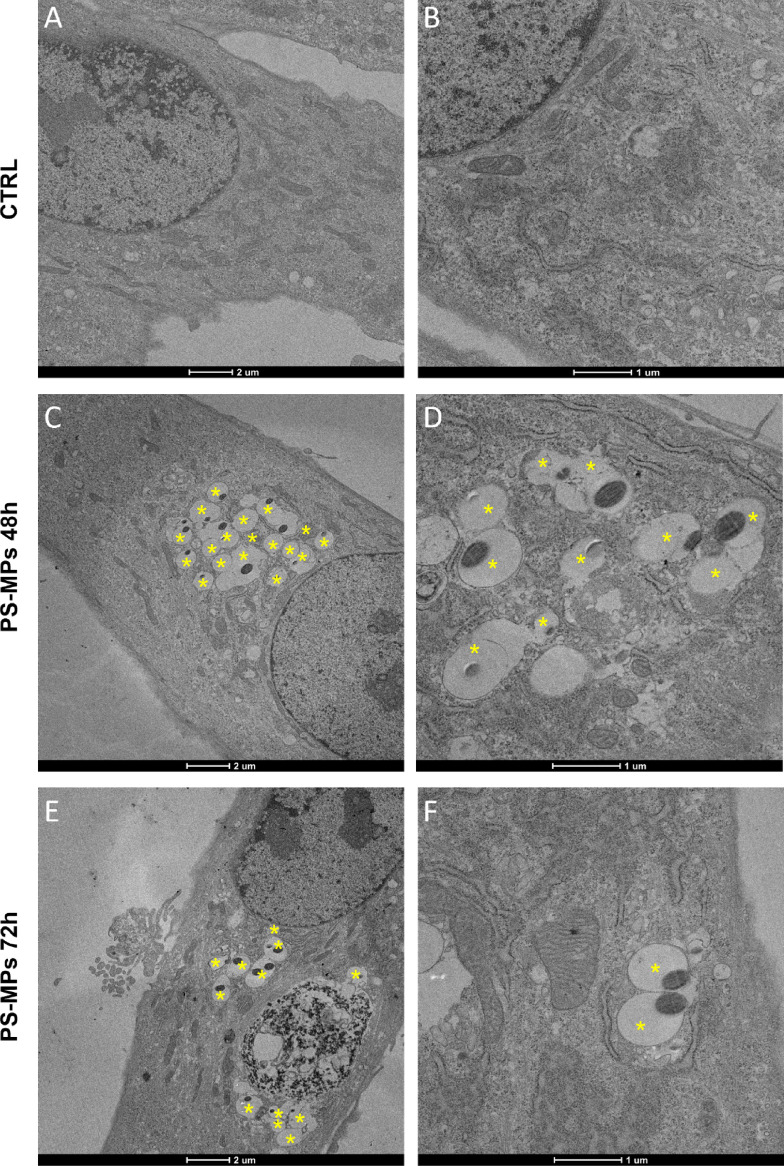
Representative TEM images of PS-MPs uptake by hGF cells. (**A**, **B**) TEM analysis of untreated hGF cells (CTRL) at 2000X magnification and 4300X magnification. (**C**, **D**) TEM analysis of hGF cells treated with 50 µg/mL 1 μm of PS-MPs for 48 h. Yellow asterisk indicates the internalized microparticles at 2000X magnification and 2600X magnification. (**E**, **F**) TEM analysis of hGF cells treated with 50 µg/mL of 1 μm PS-MPs for 72 h. Yellow asterisk indicate the internalized microparticles at 2000X magnification and 5300X magnification.

Finally, transmission electron microscopy (TEM) was used to further refine the subcellular localization of PS-MPs within hGF cells. TEM analysis showed that after 48 and 72 h, the majority of internalized PS-MPs were enclosed within intracellular membranes, likely endosomes (Fig. 3A–F). In a few TEM sections, PS-MPs were observed without a distinctly visible surrounding membrane, likely due to section orientation obscuring membrane visibility. Untreated control cells did not show any detectable presence of PS-MPs (Fig. 3A–B).

### Shotgun proteomics of hGF treated with PS-MPs

**Fig. 4:**
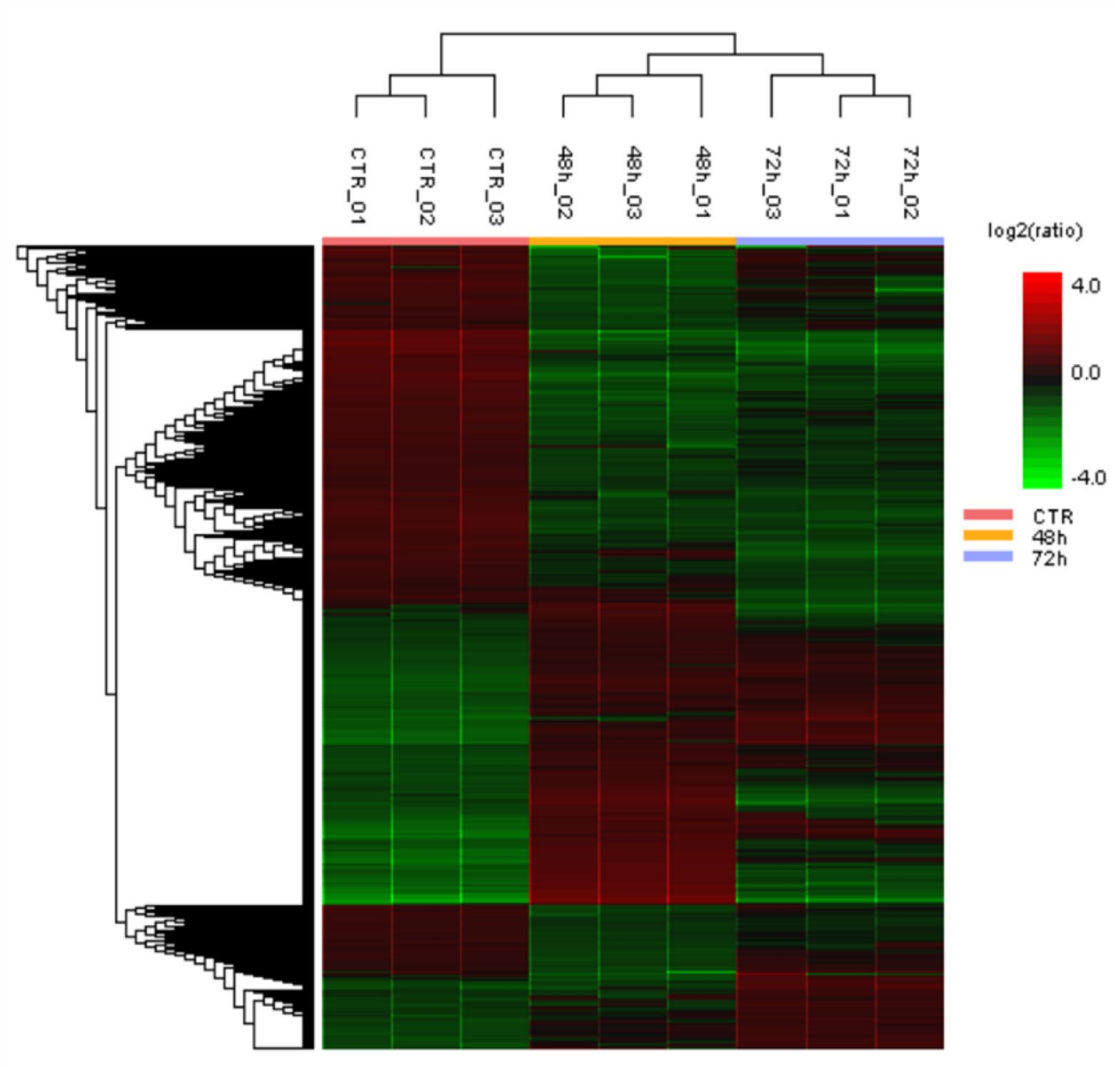
Hierarchical clustering analysis of DE proteins identified through proteomics. Hierarchical clustering analysis of differentially expressed proteins comparing CTRL (red), PS-MPs 48 h (yellow), and PS-MPs 72 h (blue) in hGF cells (3 replicates per condition). The vertical distances on each branch of the dendrogram represent the degree of similarity between protein expression profiles of different conditions. Expression levels are color-coded: red for upregulated, black for unchanged expression, and green for downregulated genes.

A shotgun label-free proteomics analysis was performed to identify proteins with altered expression following PS-MP treatment. Specifically, control and PS-MPs-treated hGF cells were lysed, and digested with trypsin according to the FASP protocol described in the “Materials and Methods” Section^29,30^. By setting the false discovery rate (FDR) at 0.5% at the peptides level, approximately 2,000 proteins were identified in each technical replicate. Label-free quantification (LFQ) analysis, using the parameters reported in the “Materials and Methods” Section, identified 389 differentially expressed (DE) proteins following PS-MPs treatment (Supplementary Tables 1, 2). As shown in Fig. 4, proteins were found to be upregulated or downregulated, displaying a consistent trend across replicates. Interestingly, some proteins were upregulated at 48 h and downregulated at 72 h, or vice versa (Fig. 4), suggesting an early and a late cellular response to PS-MPs exposure.

**Fig. 5:**
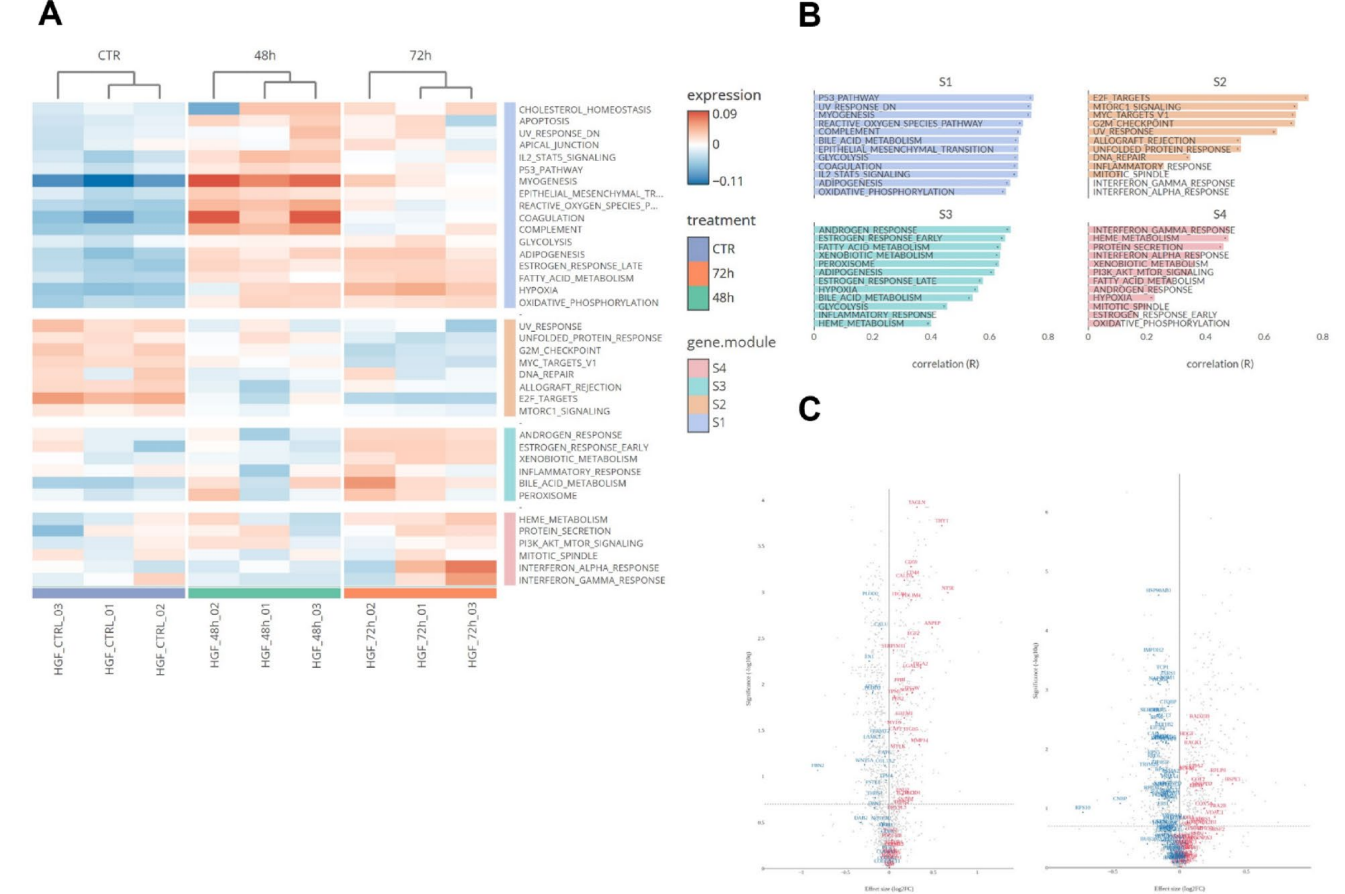
Pathway analysis of DE proteins identified through proteomics. (**A**) Clustered Heatmap displaying differentially expressed pathways in hGF cells treated with 50 µg/mL of PS-MPs for 48 and 72 h compared to control cells. (**B**) Functional annotation of the corresponding enriched gene modules based on the HALLMARK GO database. (**C**) Volcano plots illustrating significantly differentially expressed proteins associated with the EMT (left panel) and Myc (right panel) target pathways.

To gain insights into the molecular pathways and functions affected by PS-MPs treatment, the DE proteins were analysed using the BigOmics platform. Figure 5A presents a clustered heatmap of the Hallmark Gene Sets derived from the differential analysis at 48 h and 72 h compared to untreated control cells, along with the corresponding functional annotation of gene modules. Gene set 1 is mainly composed of pathways that are upregulated at both time points although mainly after 48 h of treatment (Fig. 5A). These include the epithelial-mesenchymal transition (EMT), IL-2/STAT signaling, reactive oxygen species (ROS), and various metabolic pathways such as glycolysis, adipogenesis, and oxidative phosphorylation (Fig. 5A and B). In greater detail, as illustrated in the volcano plots, EMT was represented by 66 modulated proteins, showing overall activation at both time points (Fig. 5C). Gene set 2 pathways are consistently downregulated at both time points and include E2F transcription factor targets, mammalian target of rapamycin complex 1 (mTORC1) signaling, Myc targets, and the unfolded protein response (UPR) (Fig. 5A and B). Myc targets were overrepresented by 125 modulated proteins and displayed a moderate deactivation (Fig. 5C). Gene set 3 pathways, upregulated exclusively after 72 h of treatment, includes androgen and estrogen receptor responses, fatty acid metabolism, and inflammatory responses (Fig. 5A and B). Gene set 4 pathways, upregulated exclusively after 72 h of treatment, includes interferon α and γ response and xenobiotic metabolism (Fig. 5A and B).

### Validation of key proteins/pathways identified by proteomic analysis

As described above PS-MPs treatment modulated several molecular pathways, including EMT, IL-2/STAT signalling, Myc targets, and E2F transcription factor targets. To support these inferences, we validated the expression of fibronectin, signal transducer and activator of transcription 1 (STAT1), voltage-dependent anion-selective channel protein 1 (VDAC1), and calreticulin (CALR) in hGF cells treated with PS-MPs for 48 and 72 h compared to untreated controls.

**Fig. 6:**
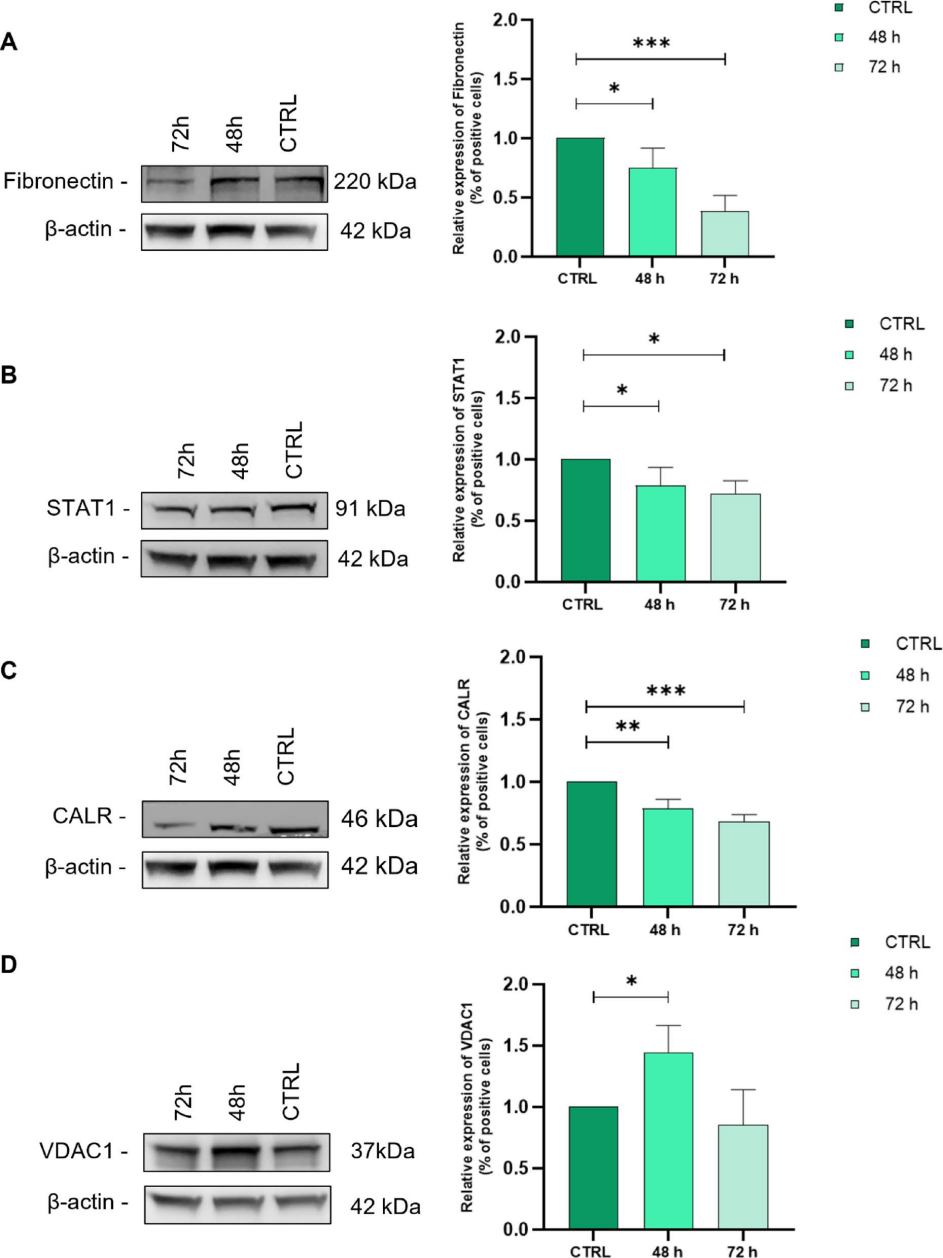
Validation of proteomic data by Western blotting. Representative Western blot analysis showing the expression of Fibronectin (**A**), STAT1 (**B**), CALR (**C**), and VDAC1 (**D**) in hGF cells after 48 and 72 h of PS-MPs treatment. Cells cultured in medium alone served as negative control (CTRL). β-actin was used as a loading reference protein. Quantification of the relative protein expression of Fibronectin (**A**), STAT1 (**B**), CALR (**C**), and VDAC1 (**D**) in hGF cells after 48 and 72 h of PS-MPs treatment, normalized to CTRL. (* *p*-value < 0.05).

Fibronectin is recognized as a reliable marker for epithelial-mesenchymal transition (EMT). In hGF cells exposed to PS-MPs, its expression showed a significant reduction at both times observed compared with untreated control cells (CTRL vs. 48 h *p*-value = 0.047; CTRL vs. 72 h *p*-value = 0.001) (Fig. 6A). STAT1 is primarily involved in the negative regulation of cell growth, differentiation, and apoptosis. Its expression showed a slight decrease at 48 h and at 72 h (CTRL vs. 48 h *p*-value = 0.048; CTRL vs. 72 h *p*-value = 0.018) (Fig. 6B). CALR is a protein involved in multiple cellular processes, like calcium homeostasis, ER stress response, and apoptosis. Its expression decreased at 48 h and 72 h (CTRL vs. 48 h *p*-value = 0.003; CTRL vs. 72 h *p*-value = 0.0003) (Fig. 6C). VDAC1 plays a crucial role in cellular metabolism and apoptosis and is associated with MYC regulation. VDAC1 levels increased after 48 h and decreased after 72 h (CTRL vs. 48 h *p*-value = 0.039) (Fig. 6D). Overall, the differential expression of these proteins in response to 1 μm PS-MPs treatment supports the proteomic findings.

**Fig. 7:**
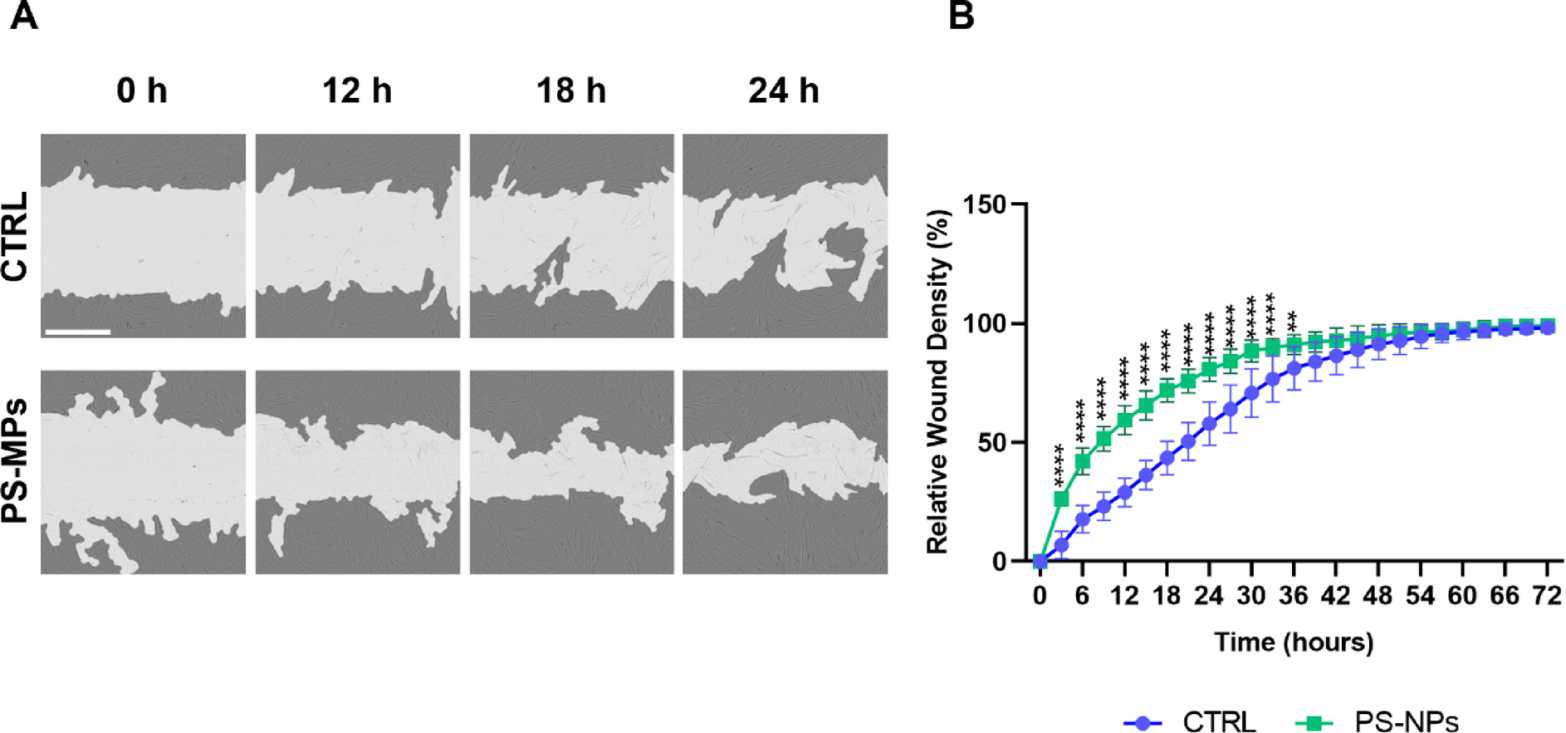
Impact of PS-MPs on the motility of hGF cells. (**A**) Representative images of the hGF cell scratch assay at baseline (0 h) and after 12, 18, and 24 h of treatment with 50 µg/mL of 1 μm PS-MPs. Scale bars represent 400 μm. (**B**) Relative wound density was measured using the Incucyte software as described in the “Materials and Methods” Section. (* *p*-value < 0.05).

Several Hallmark Gene Sets, particularly those related to EMT, suggest that PS-MP treatment may impact cell migration. To functionally validate this observation, a cell migration wound healing assay was conducted. hGF cells treated with PS-MP displayed an enhanced motility compared to untreated cells (Fig. 7A, B). For example, by 12 h post-treatment, wound closure in PS-MPs-treated hGF cells reached 59.40% ± 6.13, whereas in control hGF cells, closure was only 29.10% ± 6.00 (Fig. 7A, B). These data show a significant increase in hGF cell migration after treatment with 50 µg/ml PS-MPs, consistent with EMT involvement as indicated by the proteomic analysis.

## Discussion

Plastics have been widely recognized by the international scientific community as an emerging global threat to both environmental and human health, largely due to their pervasive accumulation in air, water, and soil. Their extensive use is driven by their versatility, durability, and low cost^22,31–33^. Plastics are composed of various polymer types, with polyethylene (PE), polypropylene (PP), polyvinyl chloride (PVC), and PS being the most common—listed here in order of global production and distribution^13,33–36^. Although PE, PP, and PVC are the most prevalent and environmentally relevant microplastics, the majority of toxicological and mechanistic studies to date have focused primarily on PS. In line with this trend, we used PS particles in our investigation. We acknowledge that this is a limitation, however, the use of PS microparticles also offers practical advantages, as they are commercially available in defined sizes and surface chemistries, which enables robust comparisons across studies and facilitates mechanistic investigations. Moreover, given the substantial body of literature using PS as a reference material, employing it allows for meaningful comparison with existing datasets.

Plastics degrade into MPs and NPs, defined as solid polymeric particles measuring between 1 μm and 5 mm (MPs), and less than 1 μm in size (NPs), respectively^31,34^. We focused on MPs due to their high prevalence in environmental samples such as oceans, soil, and air; notably, 1 μm MPs are also frequently detected in widely consumed foods and beverages^12,35–37^. Furthermore, current environmental regulations and public concern primarily focus on visible plastic pollution, which makes MP studies more immediately relevant for informing policy decisions. In contrast, NPs are more difficult to isolate and quantify, resulting in poorly established real-world exposure levels. Finally, studies involving novel cellular or tissue targets, such as gingival fibroblasts, frequently utilize MPs to explore potential biological effects and interactions, owing to the extensive availability of benchmarking data for these materials.

One of the primary routes of human exposure to microplastics is ingestion, which necessarily involves prolonged contact with oral mucosal tissues. The oral cavity is the first interface between ingested particles and human cells, and studies have shown that food intake is among the main pathways by which microplastics enter the organism and accumulate in tissues and organs^21,22^. Importantly, the gingival area represents an anatomical site where food particles, and, by extension, microplastics, can remain trapped for extended periods due to the retentive characteristics of the tissue and structures such as the gingival sulcus and periodontal pockets. Just as food debris can lodge between teeth and remain in contact with gingival tissues, MPs contained in food may persist locally, increasing the likelihood of cellular interactions and potential adverse effects. Another relevant aspect supporting the choice of this model is the widespread use of dental devices made of polymeric materials. Occlusal splints commonly used for bruxism and clear aligners employed in orthodontic treatments are composed of plastics that remain in prolonged contact with the gingiva. Wear and degradation over time can release micro- and nanoplastic particles into the oral cavity, contributing to local exposure. Therefore, studying the effects of microplastics on gingival cells is also pertinent in the context of contemporary dental practice^23^. While the gastrointestinal tract is more commonly investigated in MPs research, oral cell models offer complementary insights into local cellular responses - such as motility changes, impaired wound healing, and inflammatory activation that could have broader implications for mucosal barrier function. Since hGF are essential for maintaining oral tissue integrity and repair, alterations in their motility may contribute to pathological outcomes including delayed healing or chronic inflammation.

This study investigated the effects of PS-MPs at concentrations commonly reported in the literature (0.25–50 µg/mL)^38,39^. We would like to point out that these concentrations exceed those typically associated with chronic environmental exposure; however, high concentrations are commonly employed in toxicological studies using a high-dose, short-term approach to elicit measurable effects within a practical timeframe. Conversely, we acknowledge that certain scenarios, such as direct contact with plastic-containing materials like clothing, dental aligners or teabag, may result in locally elevated microplastic concentrations. Our data showed that 1 μm PS-MPs were not toxic to hGF cells at experimental concentrations ranging from 0.25 to 25 µg/mL after 24, 48, and 72 h of exposure, while the 50 µg/mL dose reduced cell viability. The effect of MPs on gingival fibroblasts align with existing literature, which presents mixed results: some studies report increased cytotoxicity upon microplastic exposure, whereas others observe no significant toxic effects^2,14,39,40^. The 50 µg/mL dose was selected to assess cellular effects using multiple approaches, including particle internalization. We emphasize that internalization was assessed using mildly cytotoxic concentrations of microparticles to maintain consistency with the experimental conditions applied across the study, including in the functional assays. Demonstrating efficient internalization at non-cytotoxic doses would not have been particularly informative within the time frame of our experiments.

Next, our study demonstrated that PS-MPs are internalized by hGF cells, likely via endocytosis as evidenced by the presence of intracellular membrane structures enclosing the particles. It is increasingly recognized that endocytosis plays a fundamental role in the regulation of a wide range of cellular functions^41^. Evidence that confocal microscopy and flow cytometry analyses reflect internalized rather than surface-bound MPs is supported by 3D reconstruction of confocal images, which clearly revealed the presence of MPs within the intracellular space.

We would like to highlight that both plain and amine-modified fluorescent PS microparticles were used in this study. We acknowledge that amine modification can alter the physicochemical properties of MPs and potentially influence their biological effects^12^. Importantly, all functional experiments were conducted using unmodified PS microparticles; thus, the observed biological effects can be attributed exclusively to unmodified MPs. Fluorescently labelled microparticles were used for assessing particle internalization via confocal microscopy and flow cytometry, currently the most common and straightforward methods for such analysis. While amine modification may have affected the extent of internalization, the internalization patterns observed were consistent with our TEM findings obtained using untagged microplastics. Although the level of internalisation appears low, it is consistent with what is commonly observed for MPs measuring approximately 1 µm^35,42^. Regarding the intracellular fate of internalized MPs, existing studies suggest that they tend to accumulate in the endo-lysosomal compartment^43,44^. However, as our study focused on short-term exposure and downstream molecular effects we did not examine lysosomal trafficking or long-term intracellular persistence, which represents a limitation of this work.

Next, we generated a protein expression profile for hGF cells and identified proteins and pathways influenced by PS-MPs treatment. Gene ontology analyses of these proteomic data revealed that PS-MPs treatment induced enrichment in several Hallmark GeneSets. It is worth noting that the modulation of Hallmark Gene Sets, as defined by Liberzon et al.^45^appeared more consistent than the changes observed at the individual protein level. These molecular pathways highlight the key consequences of MPs on hGF cells. Specifically, in alignment with previous studies in different cell models, they reveal alterations in cell metabolism (glycolysis, adipogenesis, and fatty acid pathways)^46,47^endocrine disruption (androgen and estrogen receptor responses)^48–50^inflammatory responses (ROS and interferons α and γ)^46,47,51^cancer progression (EMT, Myc, and UPR)^51–53^and xenobiotic metabolism^54^. Other authors demonstrated that PS-MPs reduce glycolytic activity and impair the function of antioxidant enzymes in vitro, limiting the cells’ capacity to neutralize ROS^35,55^. As a result, the cells experience significant stress and reduced ability to mitigate or eliminate harmful ROS. These findings collectively highlight the potentially damaging effects of PS-MPs on cellular function, suggesting that such damage could potentially lead to long-term adverse consequences^35^. Therefore, the excessive production of ROS can disrupt the homeostasis of cellular components, and this damage has been associated with physiological changes, genomic instability, and cancer development^40,56^. In addition, previous studies have shown that treatment with PS-NPs or PS-MPs induces the EMT process^57,58^. In our study, treatment with PS-MPs in vitro also increased migration ability by activating the EMT process. The formation of ROS, which further promotes the release of ROS from the mitochondrial respiratory chain, contributes to the EMT process. Increased ROS signalling resulted in varying degrees of mitochondrial dysfunction, which could lead to different outcomes in cell viability^57^. It is generally accepted in the literature that the phenotypic changes observed during EMT are the result of gene reprogramming involving the activation of numerous transcription factors^59^.

Among the proteins most deregulated by PS-MPs treatment, especially after 48 h of exposure, overexpression of cysteine-rich intestinal protein 1 (CRP-1) is observed. CRP-1 promotes EMT by activating the Wnt/β-catenin and GSK3/mTOR signalling pathways. Furthermore, CRP-1 interacts with gamma-butyrobetaine hydroxylase 1 (BBOX1) and STIP1 homology and U-Box containing protein 1 (STUB1) to enhance the ubiquitination and degradation of BBOX1, leading to the nuclear accumulation of β-catenin, which facilitates the induction of EMT^60^. Other proteins associated with inflammation and found to be deregulated following microplastic treatment include fibronectin, Heme Oxygenase 1 (HO-1), and Glutaredoxin-1 (GLRX1). The increase in HO-1 expression following PS-MPs treatment demonstrates that the treatment induces inflammation through oxidative stress, as this HO-1 is a key marker of such responses. Induced by oxidative stress during inflammatory processes, HO-1 likely serves as part of a cellular defence mechanism in response to stress. This induction provides negative feedback to cell activation and mediator production, potentially modulating the inflammatory response^61^. GLRX1 is a cytosolic enzyme that catalyses protein deglutathionylation. It modulates the synthesis of inflammatory mediators by regulating S-glutathionylation-sensitive signalling pathways, such as NF-κB. Overexpression of GLRX1 decreases IKKβ S-glutathionylation and enhances NF-κB activation, subsequently promoting the synthesis of inflammatory mediators^62^.

To validate our proteomics findings and provide greater strength to the above functional analysis, we confirmed the expression of a small subset of proteins (fibronectin, STAT1, calreticulin, and VDAC1) that were identified as differentially expressed. Indeed, treatment of hGFs with MPs resulted in expression changes in all four proteins that were consistent with the proteomics analysis. The reduction in fibronectin expression appears to contrast with the induction of EMT, which is typically associated with the upregulation of mesenchymal markers such as N-cadherin, vimentin, and fibronectin itself^63^. However, in some cases, EMT activation can be triggered by factors such as TGF-β, which can induce EMT through the downregulation of fibronectin levels^64^. Besides its role in EMT, fibronectin is well documented to participate in various biological processes, including cell adhesion, migration, stem cell formation, differentiation, and angiogenesis^65^underscoring the potential impact of microplastics on cell migration. Voltage-dependent anion channels (VDACs) play a critical role in cellular metabolism and the initiation of apoptosis. In cancer cells, mitochondrial VDAC1 is particularly important for promoting proliferation and migration^66^. On the plasma membrane, VDAC1 also contributes to the regulation of apoptosis, potentially through its redox activity. In addition, VDAC1 is involved in the regulation of lipid metabolism and mitophagy, processes that can trigger inflammation. Furthermore, to confirm the involvement of the JAK/STAT signalling pathway the expression of the STAT1 protein was evaluated. STAT1 is primarily involved in the negative regulation of cell growth, differentiation, apoptosis, as well as tumour initiation and metastasis^67^. Calreticulin is a chaperone protein primarily involved in protein folding and calcium buffering within the endoplasmic reticulum. Therefore, calreticulin plays a key role in maintaining proteostasis and regulating calcium-dependent processes. In specific contexts, including cancer cells, calreticulin can be translocated to the cell surface, where it influences cancer progression in a context-dependent manner. Additionally, extracellular calreticulin acts as a damage-associated molecular pattern (DAMP), contributing to inflammatory responses.

Finally, based on insights from the pathway analysis and the known roles of fibronectin, STAT1, calreticulin, and VDAC1 in regulating cell migration, we investigated whether MPs could stimulate cell motility. A wound-healing assay revealed an enhanced migratory capacity, confirming that PS-MP exposure promotes cell motility in hGF cells. In line with our findings, it has been reported that MP treatment increases the expression of adhesion molecules (VCAM-1, ICAM-1) and pro-inflammatory cytokines (IL-1β, TNF-α), changes that are associated with enhanced cell motility^68^. Additionally, a study on colorectal cancer cell lines exposed to 0.25, 1, and 10 μm PS‑MPs demonstrated a significant increase in migration only at 0.25 μm, indicating a size-dependent effect that diminishes with larger particle diameters^69–71^.

Collectively, our data indicate that alterations in specific molecular pathways regulate cell motility, adhesion, and apoptosis, with PS-MPs enhancing migration in hGF cells. Given the increasing use of plastic materials and the consequent accumulation of environmental microplastics, our findings highlight the potential adverse effects of PS-MPs on hGF cells, particularly in the context of cancer. This study is the first to investigate the impact of MPs on gingival fibroblasts and to demonstrate that exposure to PS-MPs can affect cell motility and survival. Further observational studies are necessary to elucidate the long-term consequences of PS-MPs exposure in the oral cavity and the underlying mechanisms.

## Supplementary Information

Below is the link to the electronic supplementary material.

Supplementary Material 1

Supplementary Material 2

Supplementary Material 3

## Data Availability

All data are available in the main text. Proteomics data have been submitted to the MassIVE public Repository (https://massive.ucsd.edu/) and are accessible under the MassIVE identifier MSV000096484 and ProteomeXchange identifier PXD058127.
