# Supplementary material for: Polystyrene microplastics are internalized by human gingival fibroblasts, enhance cell motility and induce molecular changes revealed through proteomic analysis
Source: Sci Rep. 2025 Oct 8;15:35128. doi: 10.1038/s41598-025-19064-w (PMC12508049; doi:10.1038/s41598-025-19064-w)

SUPPLEMENTARY INFORMATION II SOURCE DATA

Polystyrene Microplastics can be endocytosed by Human Gingival Fibroblasts and Enhance Cell Motility: additional functional insights from proteomic analysis

Federica Di Cintio[1,2], Anna Giulia Ruggieri[2,3], Chiara De Simone[1], Piero Di Carlo[2,3], Maurizio Ronci[1,2*], Vittoria Perrotti[3,4,*] and Michele Sallese[2,3,*]

[1]Department of Oral, Medical and Biotechnological Sciences, University “G. d’Annunzio” of Chieti-Pescara, Chieti, Italy.

[2] Center for Advanced Studies and Technology (CAST), University “G. d’Annunzio” of Chieti-Pescara, Chieti, Italy.

[3]Department of Innovative Technologies in Medicine & Dentistry, University “G. d’Annunzio” of Chieti-Pescara, Chieti, Italy.

[4] UdA-TechLab, Research Center, “G. d’Annunzio” University of Chieti-Pescara, Via dei vestini, 31-66100 Chieti, Italy; Chieti, Italy;

*Correspondence: Maurizio Ronci, Vittoria Perrotti, Michele Sallese

Email: maurizio.ronci@unich.it, vittoria.perrotti@unich.it, michele.sallese@unich.it

Figure 6A – Validation of proteomic data by Western blotting.

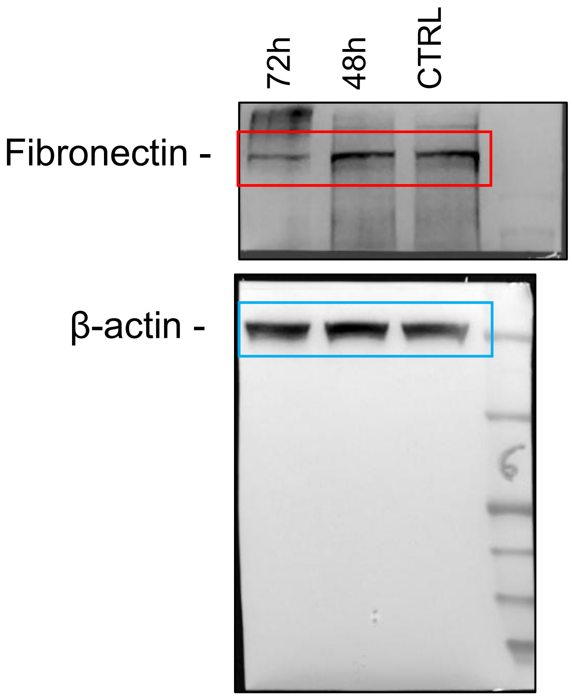

Figure 6B – Validation of proteomic data by Western blotting.

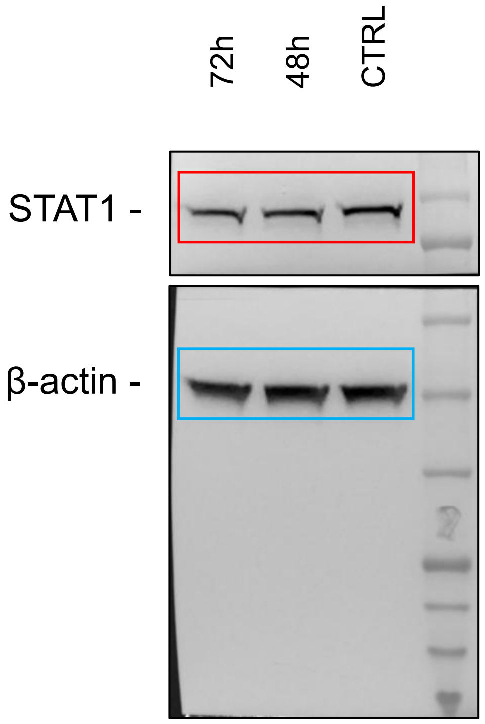

Figure 6C – Validation of proteomic data by Western blotting.

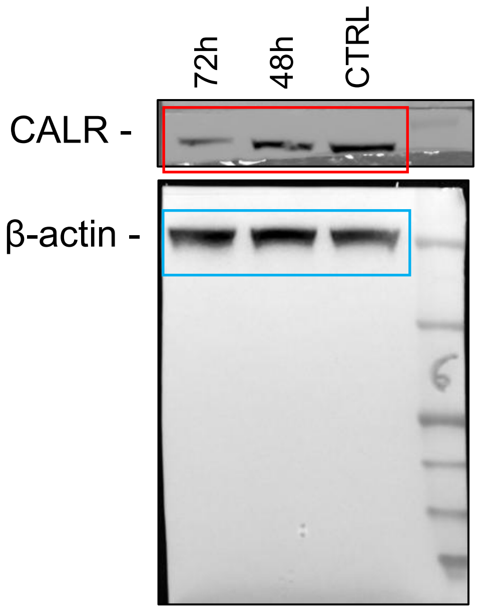

Figure 6D – Validation of proteomic data by Western blotting.

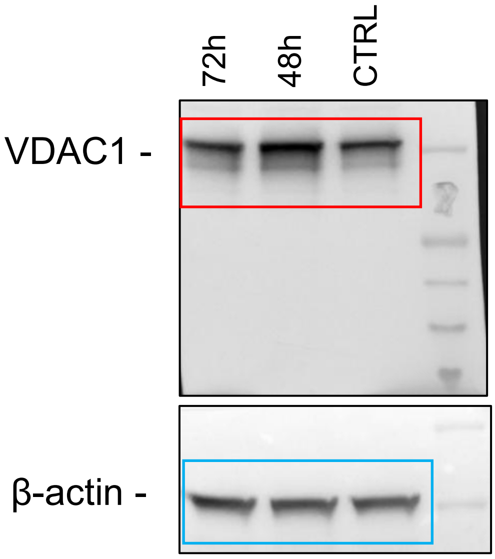

Supplement: Supplementary file 1 — Supplementary Material 1 [file 41598_2025_19064_MOESM1_ESM.pdf]
